# Prolonged *Bartonella henselae* Bacteremia Caused by Reinfection in Cats

**DOI:** 10.3201/eid1401.070768

**Published:** 2008-01

**Authors:** Mardjan Arvand, Juliane Viezens, Julia Berghoff

**Affiliations:** * University of Rostock, Rostock, Germany

**Keywords:** Bartonella henselae, bacteremia, cat, relapse, reinfection, dispatch

## Abstract

We analyzed the genetic relatedness of blood culture isolates of *Bartonella henselae* from 2 cats of patients with cat-scratch disease at admission and after 12 months. Isolates from each cat at different times were clonally unrelated, which suggested reinfection by a second strain.

*Bartonella henselae* is a zoonotic pathogen associated with a broad spectrum of disease manifestations in humans. Cat-scratch disease (CSD) is commonly encountered in immunocompetent patients; in immunocompromised patients, bacillary angiomatosis, peliosis hepatis, and recurrent bacteremia are usually seen. Domestic cats represent the main host and reservoir for *B*. *henselae* (*1*). Recurrent, intraerythrocytic bacteremia develops in infected cats without overt clinical symptoms (*2*). Experimental infection of specific pathogen–free cats with *B*. *henselae* induces recurrent episodes of bacteremia, which, in most cases, resolve spontaneously within 22–33 weeks postinfection (*3*–*5*). Prolonged bacteremia >7 months has been documented sporadically, e.g., in 1 of 12 experimentally infected cats inoculated with the highest infectious dose (this cat was bacteremic 32 weeks postinfection) (*3*), or in an unspecified number of cases in 21 experimentally infected cats that were bacteremic 48 weeks postinfection (*5*).

Few studies have investigated the course of recurrent bacteremia in naturally infected cats because follow-up investigations are difficult to conduct (*1*,*6*–*9*). Koehler et al. (*1*) detected recurrent bacteremia with a duration >2 months in 3 cats of patients with bacillary angiomatosis. Kordick et al. (*6*) reported positive blood cultures in cats of several CSD patients up to 14 months after collection of the initial positive culture. In the latter study, the first blood culture was collected from the index cat of 1 CSD patient 22 months after the onset of the disease in the patient and contained *B*. *henselae* (*7*). Sander et al. (*8*) found repeated bacteremia in the cat of a CSD patient after 5 months and in 2 other cats after 1 year. In another study, *B*. *henselae* was isolated from the blood culture of a cat of a patient who had an episode 2.5 years earlier of debilitating fatigue with a duration of 1 month and without fever or lymphadenopathy (*7*). *B*. *henselae* was isolated again from the blood culture of the index cat after 5 months (*7*).

In these studies, the question whether the cats were still infected by the initial *B*. *henselae* strain or had acquired a new strain was not addressed. It was concluded that the cats were persistently infected with *B*. *henselae* (*8*). We have recently demonstrated the appropriateness of pulsed-field gel electrophoresis (PFGE) and multilocus sequence typing (MLST) for differentiation of *B*. *henselae* isolates to the strain level (*10**,**11*). Therefore, we analyzed the clonal relationship between sequential *B*. *henselae* isolates that were obtained at different times from the blood of 2 cats to determine whether recurrences were caused by the initial strain or a new strain.

## The Study

We tested 4 isolates of *B*. *henselae*: FR96/BK36, FR96/BK36II, FR96/BK75, and FR96/BK75II. These isolates were grown from the blood of 2 naturally infected cats (cat 36 and cat 75) of CSD patients at first consultation and after 12 months, respectively (*9*). The original colony counts were 100, 100, 120, and 100, respectively. PFGE analysis was conducted after digestion of DNA with *Sma*I, and MLST was conducted after partial sequencing of 8 genetic loci (*12**,**13*). PFGE analysis showed 9 band differences between isolates 36 and 36II and 10 band differences between isolates 75 and 75II (Figure), which suggested that isolates obtained from the same cat at different times were not clonally related (*14*). MLST analysis showed 3 and 6 different alleles between isolates 36 and 36II and isolates 75 and 75II, respectively (Table). Isolates 36 and 36II were assigned to sequence type (ST) 14 and ST5, which have the 16S rRNA alleles 1 and 2, respectively. Isolates 75 and 75II were assigned to ST5 and ST7, respectively, and both had the 16S rRNA allele 2 (Table). These data suggest that both cats were infected by a second *B*. *henselae* strain at the second time blood was obtained.

**Figure:**
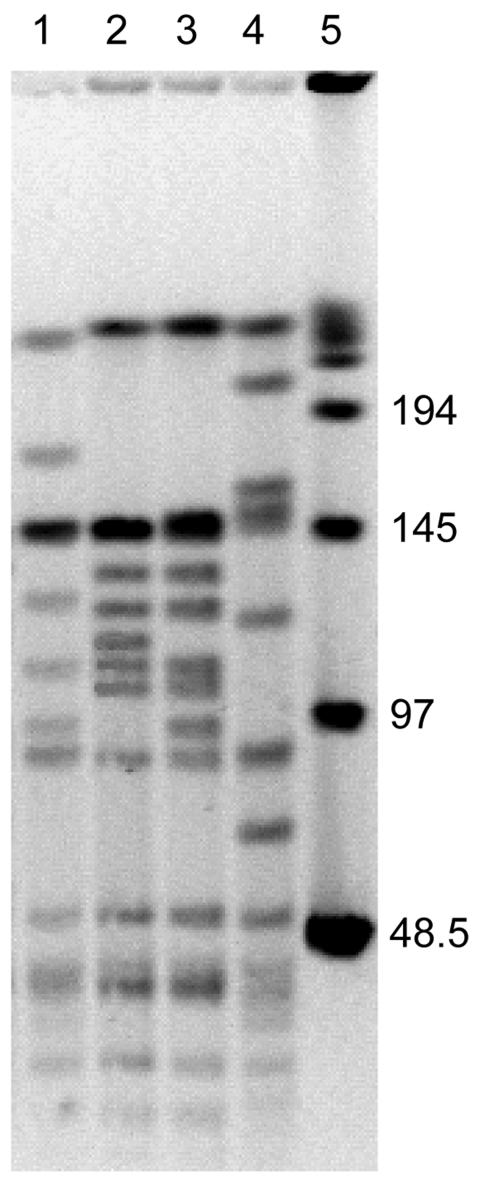
*Sma*I macrorestriction patterns of *Bartonella henselae* isolates from 2 cats. Lane 1, cat 36, first isolate; lane 2, cat 36, second isolate obtained 12 months later; lane 3, cat 75, first isolate; lane 4, cat 75, second isolate obtained 12 months later; lane 5, bacteriophage λ molecular mass pulsed-field gel electrophoresis marker. Values on the right are in kilobases.

**Table:** Multilocus sequence typing data of 8 genetic loci of 4 *Bartonella henselae* isolates from 2 naturally infected cats*

Isolate	16S rRNA	*batR*	*gltA*	*ftsZ*	*groEL*	*nlpD*	*ribC*	*rpoB*	ST
FR96/BK36	1	2	1	2	2	1	1	1	14
FR96/BK36II	2	1	1	1	2	1	1	1	5
FR96/BK75	2	1	1	1	2	1	1	1	5
FR96/BK75II	2	4	2	3	1	2	2	1	7

*Values are allele numbers. ST, sequence type.

## Conclusions

Our data indicate that recurrent *B*. *henselae* bacteremia in naturally infected cats does not necessarily represent a relapse but may be caused by reinfection. We were surprised to find that both cats were presumably reinfected by a different strain within 1 year. The interval between collection of the initial and follow-up blood samples was long, which might explain the high rate of reinfection. The possibility that the cats were infected by 2 *B*. *henselae* stains at the time blood was first obtained was examined by subjecting 5 single-colony–derived cultures of each initial isolate to PFGE analysis, which did not show evidence for co-infection. In addition, co-infection by 2 strains is unlikely because we would not have been able to determine a unique sequence for those genetic loci that displayed allelic polymorphism. Furthermore, co-infection by 2 strains in both cats would represent a rare coincidence. Nevertheless, we cannot completely rule out co-infection with 2 strains at the time blood was first obtained, with 1 strain being highly underrepresented among initial colonies.

To our knowledge, this is the first report to document sequential infection by different *B*. *henselae* strains in naturally infected cats by using 2 independent typing methods. In a previous study, Kabeya et al. (*9*) reported variations of up to 5 bands in PFGE patterns of *B*. *henselae* isolates obtained from naturally infected cats during different bacteremic peaks. However, we have recently demonstrated that genetic variants displaying 1- to 4-band differences frequently occur within primary *B*. *henselae* isolates and do not necessarily indicate infection by a different strain (*11*,*12*). Careful interpretation of PFGE typing results, use of additional restriction endonucleases, or use of other typing techniques is necessary to ensure correct classification of different patterns to the variant or strain level. *B*. *henselae* isolates collected from different bacteremic episodes of naturally infected cats after a long interval should by tested by molecular typing to determine their clonal relatedness.

In conclusion, our data emphasize the requirement for molecular typing to differentiate between relapse and reinfection by *B*. *henselae* in naturally infected cats. Studies on additional isolates are required to evaluate the frequency of reinfection by a different strain in naturally infected cats. Results of these studies would provide a better understanding of the natural course of feline infection. Our data also suggest that infection by a distinct *B*. *henselae* strain does not induce protective immunity against subsequent infection by a clonally unrelated strain. These results are partially consistent with those of Yamamoto et al. (*15*), who found incomplete cross-protection between isolates with different 16S rRNA alleles. Recent studies have shown that the delineation of *B*. *henselae* isolates into 2 genotypes on the basis of 16S rRNA sequence is not consistent with phylogenetic classifications using other genetic loci and does not reflect clonal lineage of isolates (*11**,**14*). Reevaluation of induction of cross-protection between different *B*. *henselae* strains in an experimental infection model is needed.
